# A multi-country study of the economic burden of dengue fever based on patient-specific field surveys in Burkina Faso, Kenya, and Cambodia

**DOI:** 10.1371/journal.pntd.0007164

**Published:** 2019-02-28

**Authors:** Jung-Seok Lee, Vittal Mogasale, Jacqueline K. Lim, Sowath Ly, Kang Sung Lee, Sopheak Sorn, Esther Andia, Mabel Carabali, Suk Namkung, Sl-Ki Lim, Valéry Ridde, Sammy M. Njenga, Seydou Yaro, In-Kyu Yoon

**Affiliations:** 1 International Vaccine Institute, Seoul, South Korea; 2 Institute Pasteur, Phnom Penh, Cambodia; 3 Kenya Medical Research Institute (KEMRI), Nairobi, Kenya; 4 McGill University, Montreal, Quebec, Canada; 5 French Institute for Research on Sustainable Development (IRD), Universités Paris Sorbonne Cités, Paris, France; 6 University of Montreal Public Health Research Institute (IRSPUB), Montreal, Canada; 7 Centre MURAZ, Bobo-Dioulasso, Burkina Faso; University of Heidelberg, GERMANY

## Abstract

**Background:**

Dengue fever is a rapidly growing public health problem in many parts of the tropics and sub-tropics in the world. While there are existing studies on the economic burden of dengue fever in some of dengue-endemic countries, cost components are often not standardized, making cross-country comparisons challenging. Furthermore, no such studies have been available in Africa.

**Methods/Principal findings:**

A patient-specific survey questionnaire was developed and applied in Burkina Faso, Kenya, and Cambodia in a standardized format. Multiple interviews were carried out in order to capture the entire cost incurred during the period of dengue illness. Both private (patient’s out-of-pocket) and public (non-private) expenditure were accessed to understand how the economic burden of dengue is distributed between private and non-private payers.

A substantial number of dengue-confirmed patients were identified in all three countries: 414 in Burkina Faso, 149 in Kenya, and 254 in Cambodia. The average cost of illness for dengue fever was $26 (95% CI $23-$29) and $134 (95% CI $119-$152) per inpatient in Burkina Faso and Cambodia, respectively. In the case of outpatients, the average economic burden per episode was $13 (95% CI $23-$29) in Burkina Faso and $23 (95% CI $19-$28) in Kenya. Compared to Cambodia, public contributions were trivial in Burkina Faso and Kenya, reflecting that a majority of medical costs had to be directly borne by patients in the two countries.

**Conclusions/Significance:**

The cost of illness for dengue fever is significant in the three countries. In particular, the current study sheds light on the potential economic burden of the disease in Burkina Faso and Kenya where existing evidence is sparse in the context of dengue fever, and underscores the need to achieve Universal Health Coverage. Given the availability of the current (CYD-TDV) and second-generation dengue vaccines in the near future, our study outcomes can be used to guide decision makers in setting health policy priorities.

## Author summary

Dengue fever is a major public health concern in many parts of South-East Asia and South America. In addition to countries where dengue has been highly prevalent for many years, there is a growing concern on the undocumented burden of dengue in Africa. Following the successful execution of the first-round economic burden study in Vietnam, Thailand, and Colombia by the Dengue Vaccine Initiative, the second-round economic burden study was implemented in Burkina Faso, Kenya and Cambodia using the same standardized methodology. In particular, the second-round study targeted GAVI eligible countries for future vaccine introductions and included two African countries where the burden of dengue was relatively unknown. Our study outcomes show that the economic burden of dengue fever is significant in all three countries. The dengue vaccination era began in 2016 with the first dengue vaccine (CYD-TDV) although its public use should be carefully determined due to the safety concerns related to the vaccine. Considering that there are other second-generation dengue vaccines in development, the current study outcomes provide an important step to estimate the economic benefits of vaccination in the three countries.

## Introduction

Dengue fever is a vector-borne disease and transmitted by *Aedes* mosquitoes [1–3]. Dengue immunity and population biology are complex [4,5]. There are four serotypes, which are antigenically distinct viruses but interact with each other. It is known that infection with one serotype provides life-long protection against that specific serotype, but a subsequent heterotypic infection may lead to favorable (short-term cross protection) or detrimental (the development of more severe illness) outcomes due to a high degree of antigenic cross-reactivity [5–7]. Despite continuous efforts to disentangle the complexity of the disease, it is still not clear how all four serotypes interact with each other in terms of cross protection, antibody dependent enhancement (ADE), and the duration of the serotype interactions [8,9].

The complex nature of the disease also imposes difficulties on the development of safe and effective dengue vaccines. A first live-attenuated, tetravalent dengue vaccine (Dengvaxia, CYD-TDV) became commercially available in 2016, but the safety concerns related to the vaccine have created a wide range of controversial debates [10–13]. Such challenges for developing safe and effective dengue vaccines have been a part of the reasons why there has been relatively less attention paid to the health-economic aspect of the disease.

As previously mentioned by Lee et al. [14], a relatively small number of empirical economic burden studies of dengue are available. Some of the existing studies relied on secondary data sources and extrapolated to other countries given a lack of field-based datasets. While Suaya et al. and Lee et al. conducted the economic burden study of dengue fever in a multi-country setting based on primary data sources using standardized methods [14,15], many of other studies applied different study designs and methodologies, making it difficult to make proper comparisons across countries. There is no doubt that all of the existing studies have contributed to informing the importance of the economic burden of dengue fever, but it is also true that more field-based studies with standardized methods are essential to better understand the economic burden of dengue fever in many of known and unknown dengue-endemic countries.

In order to fill the existing knowledge gaps, the Dengue Vaccine Initiative (DVI) implemented multi-disciplinary cost-of-illness (COI) field surveys in six countries in collaboration with research partners. As a first round of the project, the economic burden study had been carried out in Vietnam, Thailand, and Colombia from 2012 to 2015, and the final study outcome was recently published [14]. Following the successful execution of the first-round surveys, the DVI expanded the COI field studies to three additional countries: Burkina Faso, Kenya, and Cambodia. Considering that the first dengue vaccine was about to be available when the second-round countries were being selected, GAVI eligibility was also taken into account for vaccination in the future. In particular, the second-round field surveys included two countries in Africa where dengue burden is relatively unknown compared to other tropical and sub-tropical countries in South Asia and Latin America.

Understanding the accurate economic burden of a disease is one of the important steps to grasp a full scope of vaccination benefits from the societal perspective. As previously shown, the range of the total COI for dengue fever plays a critical role in determining the threshold costs for which dengue vaccination would be effective [16]. Considering that there are several second-generation vaccine candidates which are currently in phase 3 trials, the current economic burden study would contribute to filling the knowledge gaps in Burkina Faso, Kenya, and Cambodia where healthcare resources are limited, and healthcare budget may be highly constrained considering several competing health problems.

## Methods

Ideally, the COI study would be conducted in an area where dengue transmission is prevalent. This may not be an issue for many of South Asian countries such as Cambodia where the prevalence of dengue fever has been known for many years. However, appropriate site selection was a challenge in Burkina Faso and Kenya because there was a lack of information regarding dengue fever during the initial phase of the study. Thus, study sites in these two countries were selected based on their likelihood of supporting dengue transmission using outbreaks and case reports in the literature in Africa. The full description is available in the study by Lim et al. [17].

Table 1 shows information on study sites. Similar to the first round COI study, the current COI study was embedded into ongoing dengue epidemiological field studies. In Burkina Faso, five centers for health and social advancement (CSPS) located in Ouagadougou were selected. The CSPS are the first-level health system facilities which provide basic healthcare and medical resources for local populations. The CSPS consists of three units: Expanded Program of Immunization (EPI), gynecology and obstetrics, and general medicine. The facility has examination rooms, inpatient wards (where patients can stay up to three days), and staff offices. In Kenya where health facilities are categorized into six levels, three health facilities were selected in Mombasa: Ganjoni, Coast Provincial General Hospital (CPGH), and Tudor. Ganjoni is a community-level health service provider (level 1) and mainly provides services for outpatients with limited health services. Tudor sub-county hospital is a district-level healthcare provider (level 3). The facility has 14 beds and provides various services including inpatient department care, family planning, anti-retroviral therapy, as well as home-based care. CPGH is the second largest governmental hospital and serves as a tertiary referral center (level 5). The hospital has about 700 beds with approximately 800 staff members. In Cambodia, the COI study was implemented in two provincial-level and two district-level health facilities in three provinces: Kampong-Cham, Tbong-Khmum, and Kampot. The district- and provincial-level health facilities in Kampong-Cham have 30 beds with 32 staff and 260 beds with approximately 250 health workers, respectively. The health facility in Tbong-Khmum, which was separated out from Kampong Cham province, is a district-level facility with 90 beds and 59 staff. Another provincial-level facility in Kampot province is staffed with 95 health workers and maintains 155 beds.

**Table 1:** Study sites.

Country	Province / city	Study period	Study facility
Burkina Faso	Ouagadougou	Jun, 2015—Feb, 2017	CSPS de Pazani CSPS de Sect 22 CSPS de Sect 25 CSPS de Zongo CSPS de Juvénat Fille
Kenya	Mombasa	Apr, 2016—May, 2017	Ganjoni CPGH Tudor
Cambodia	Kampong-Cham Tbong-Khmum Kampot	Jul, 2015—Oct, 2016	Kampong-Cham Referral health facility Kampong-Cham Provincial health facility Tbong-Khmum Referral health facility Kampot Provincial health facility

As one of the main goals of the DVI study was to estimate the economic burden of dengue fever by using a standardized method across the sites, the current COI surveys were implemented following the similar methodologies applied to the first-round countries. The detailed study design and overall structure were fully described by Lee et al. [14].

Briefly, patients experiencing fever for less than 7 days were recruited for the fever surveillance, and rapid tests (NS1, IgM/IgG) were implemented. Due to the slow caseload during the first-half of the study period in Cambodia, the polymerase chain reaction (PCR) was additionally used to meet the desired sample size. Among those who were positive on any of the test results and consented to the study, the economic burden survey was carried out. Multiple interviews were conducted up to three times depending upon the duration of illness in order to capture the entire costs of the current dengue illness: costs spent before the study enrollment visit, during the study enrollment visit, and after the enrollment visit (S1 Text). The target sample size was estimated to be approximately 150 patients for each study site. The simple random sampling method was applied [18], where an acceptable difference between a true population and a sample estimate was assumed to be 0.2 with the 95% confidence interval statistic (= 1.96). The coefficient of variation was obtained from an existing multi-country study [15].

The COI survey included three major cost components: direct medical costs (DMC), direct non-medical costs (DNMC), and indirect costs (IC). DMC consists of consultation fees, medication, laboratory tests, and all other costs which are directly related to the medical treatment of the current dengue illness. Patients were asked how much money they spent for medical services that they received, and whether they had to bear all of the expenditure directly or were covered by any external supports such as private/public insurance, government subsidies, or non-governmental aids. In order to capture the full spectrum of the DMC, hospital bill records were also accessed to understand how the DMC burden was distributed between private and non-private payers. DNMC includes all expenditure spent for food, lodging, and transportation for a patient as well as the patient’s accompanies.

IC takes account of the costs of productivity loss (i.e. wage loss, missing school days), substitute laborers, and caretakers. In order to estimate productivity loss, the self-reported daily wage loss was asked for patients who make earnings. For students who do not earn any wages, the government expenditure per primary student expressed in 2015 USD (2014 USD in Cambodia due to data availability) was used to convert their productivity loss into monetary value. If a patient was neither a wage-worker nor a student (i.e. unpaid housework), the minimum wage of each country was applied. While the government expenditure per primary student is useful for comparing average spending on one student between countries, the use of this indicator may underestimate their productivity loss as the indicator does not include household contributions [14].

In addition to productivity loss, patients were also asked whether they had hired any substitute laborers or caretakers during their illness. If yes, a series of questions related to the duration and payments of having substitute laborers and/or caretakers were asked. In case that patients did not pay anything for having them (i.e. household members), the opportunity costs of substitute laborers/caretakers were estimated by taking into account the daily payments for doing their usual activities which they would have done otherwise. It should be noted that the questionnaire was carefully designed in order to avoid any duplication of the costs. In other words, patient’s productivity loss was not double counted when combining patient’s wage loss with substitute laborer(s)’ costs. The detailed study design which avoids the duplication of productivity loss estimation was fully addressed by Lee et al [14].

In many circumstances, costs may not be the same as charges for various reasons as indicated by previous studies [14,19,20]. While estimating economic burden using hospital charge information reflects better on patients’ direct burden, adjusting hospital charges by the ratio of cost-to-charge (RCC) is another way to understand the overall societal cost of a disease [19,20]. As previously defined, RCC was estimated by dividing the overall annual hospital costs by the total hospital revenue of the hospital [14]. In Burkina Faso and Kenya, the RCC was estimated for each study facility by accessing the annual financial reports which provide comprehensive revenue sources and expenditure types of the health facilities: external funding, additional services, labor costs (staff salary, welfare), material costs, and capital assets, etc. On the other hand, the study team was not able to access the full scope of financial reports for the health facilities in Cambodia due to logistical issues. The study facilities in Cambodia charged patients a package (uniformed) price, which covers a range of medical services such as consultation and medication. Given the limitations, the medical service utilization form was additionally implemented to collect unit costs and quantities of medical services in Cambodia. Other health facility costs such as staff salary, materials, and electricity, etc. were separately obtained. The overall economic burden was presented from both the hospital-charge and societal-cost perspectives. Taking into account the skewed distribution of cost data in general [21,22], bootstrapping was conducted to generate a 95% confidence interval with the percentile method (2.5^th^ and 97.5^th^ percentiles of the distribution) [8]. All estimates were expressed in 2016 USD using the official exchange rate from the World Bank, as well as the purchasing power parity (PPP).

Given that dengue burden is relatively unknown in Burkina Faso and Kenya, we investigated the understanding of the disease in the general public. Thus, the dengue perception score was constructed by combining the following factors: whether a respondent is aware of (1) how dengue is transmitted, (2) proper ways to get treated when infected, and (3) best ways to avoid dengue. The perception score was ranged between 1 and 3 where a higher number indicates more knowledge on dengue fever. In addition, respondents were also asked about their monthly household income, and the total out-of-pocket expenditure of dengue fever was estimated as a proportion of household monthly income to understand the extent of the direct economic burden borne by patients due to dengue infection. Households were categorized into three income groups based on percentiles of monthly household income reported by respondents: low-income group (income≤25%), middle-income group (25%<income≤75%), and high-income group (income>75%). Respondents who did not report their monthly income were categorized into the three income groups by comparing their levels of household-assets with the ones for the respondents who reported income [14].

### Ethics statement

The cost-of-illness studies were approved by the Institutional Review Boards (IRB) of the International Vaccine Institute, as well as by the ethical review committees of host country institutions: the IRB of the Centre Hospitalier de l’Universitede Montreal (CRCHUM) in Canada and the National Health Ethical Committee in Burkina Faso, KEMRI Scientific and Ethical Review Unit and the Ethical Review Committee of CPGH in Kenya, and the National Ethics Committee for Health Research (NECHR) in Cambodia. All patients who were enrolled into the COI studies completed the written informed consent form. For minors under the age of 18 years old, their parents or guardians were asked to provide consent on behalf of their children.

## Results

Table 2 summarizes descriptive statistics. The total number of patients enrolled in the study was the highest in Burkina Faso (n = 414) due to the dengue outbreak occurred during the study period in Ouagadougou. In Cambodia, all dengue-probable cases were automatically hospitalized, thus there was no outpatient enrolled. On the other hand, inpatients were not included because of logistical issues in Kenya. The average number of sick days ranged from 6 to 9 days. Patients tended to have more caretakers than substitute laborers during their illness. While on average, a majority of inpatients were completely unable to perform their usual activities during their illness in Cambodia, patients in Burkina Faso and Kenya were at least partially able to carry out their usual activities during the half of the total sick period. The mean age of patients was lower in Cambodia compared to that in Burkina Faso and Kenya, which in turn, results in the higher proportion of patients studying in Cambodia (see S1 Table for additional information). The average monthly household income was higher in Burkina Faso than in Kenya and Cambodia. Among respondents, dengue vector control activities were more common in Burkina Faso and Cambodia than in Kenya. Types of health facilities where patients visited before and after the study enrollment are further summarized in S1 Fig.

**Table 2:** Descriptive statistics.

Item	Burkina Faso		Kenya		Cambodia	
	Inpatient	Outpatient	Inpatient	Outpatient	Inpatient	Outpatient
N	141	273	-	149	254	-
Number of sick days prior to enrollment (mean, SD)	3.7 (1.0)	2.8 (1.2)	-	3.0 (1.8)	3.4 (1.7)	-
Number of sick days (mean, SD)	6.2 (2.2)	5.7 (1.9)	-	8.1 (3.2)	8.6 (2.7)	-
Proportion of patients with substitute laborers (SD)	8.1 (0.3)	5.9 (0.2)	-	21.3 (0.4)	8.6 (0.3)	-
Proportion of patients with caretakers (SD)	30.5 (0.5)	16.8 (0.4)	-	73.2 (0.4)	100.0 (0.0)	-
Number. of full days lost due to illness^a^ (mean, SD)	2.9 (2.4)	2.6 (1.8)	-	4.1 (2.9)	7.0 (2.7)	-
Number. of partial days lost due to illness^a^ (mean, SD)	2.8 (1.4)	2.5 (1.4)	-	2.9 (2.4)	1.1 (1.5)	-
Patient age (mean, SD)	25.8 (12.6)	27.7 (12.0)	-	23.9 (8.4)	10.4 (5.6)	-
Proportion of patients studying (SD)	43.3 (0.5)	33.7 (0.5)	-	48.3 (0.5)	66.9 (0.5)	-
Proportion of patients working (SD)	34.0 (0.5)	36.3 (0.5)	-	33.6 (0.5)	33.6 (0.5)	-
Monthly household income (mean, SD)	$385 (298.2)	$372 (265.9)	-	$252 (226.8)	$245 (415.1)	-
Proportion of respondents with vector control activities (SD)	99.5 (0.1)		61.1 (0.5)		97.6 (0.2)	

^a^ The number of full / partial days lost is for those older than 5 years old.

**Fig 1:**
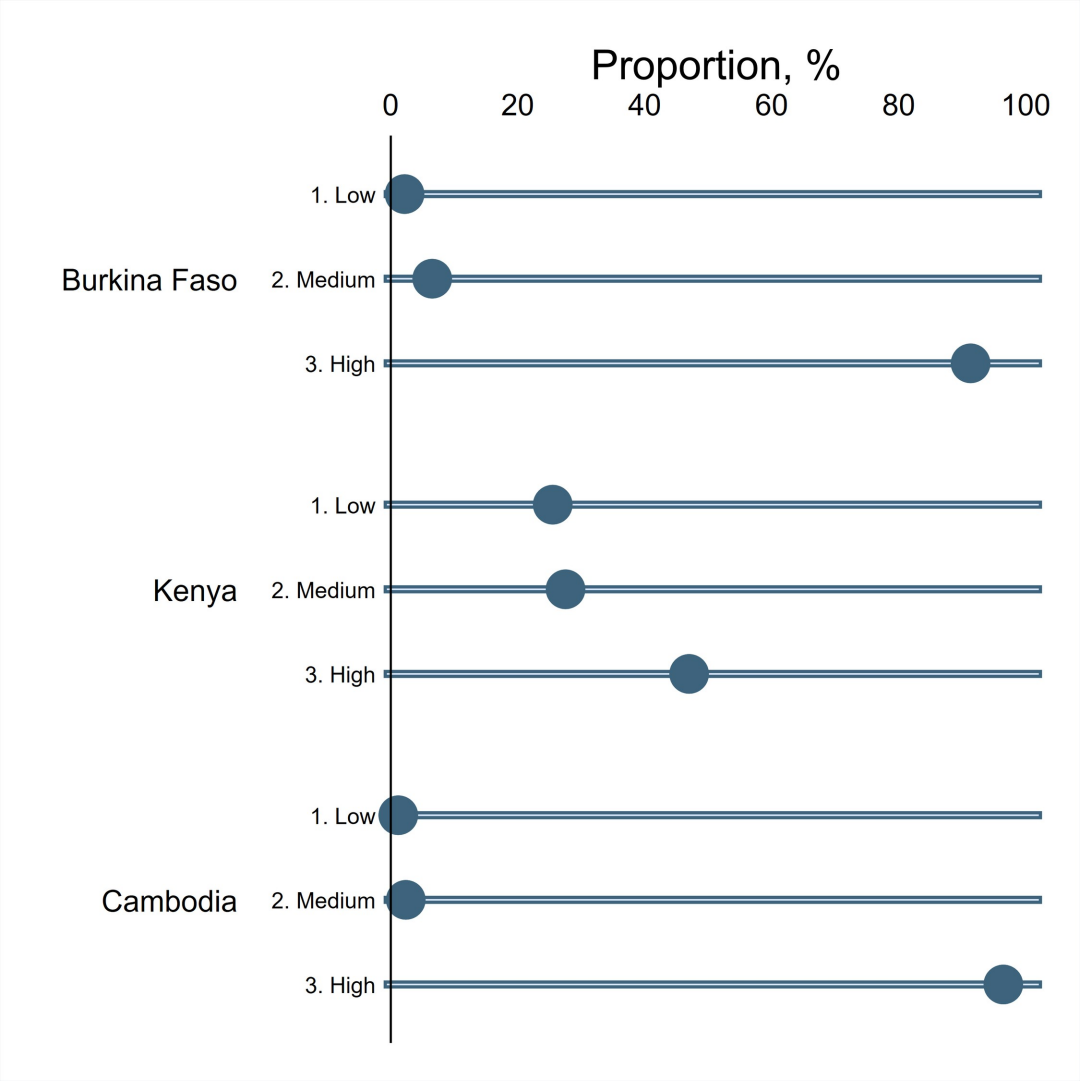
Dengue perception. Fig 1 demonstrates the level of dengue awareness reported by respondents. The perception score was divided into three categories, and the proportion of each category shows the relative size of respondents who belong to that category.

Dengue awareness was quantified by constructing the dengue perception score as shown in Fig 1. As dengue has been prevalent for many years in Cambodia, over 95% of the respondents were well aware of the disease in Cambodia. It is interesting to see that a majority of the respondents fell into the highest category of the perception score in Burkina Faso although the percentage is lower than that of Cambodia. This high perception score observed in Burkina Faso may have been due in part to the dengue outbreaks occurred during and before the study period [23]. In contrast, less than 50% of the respondents scored the highest number in Kenya, reflecting that dengue was a relatively unknown disease to the general public compared to the other two countries. The low-level perception score in Kenya might be related to the fewer number of respondents who conducted vector control activities as shown in Table 1.

**Fig 2:**
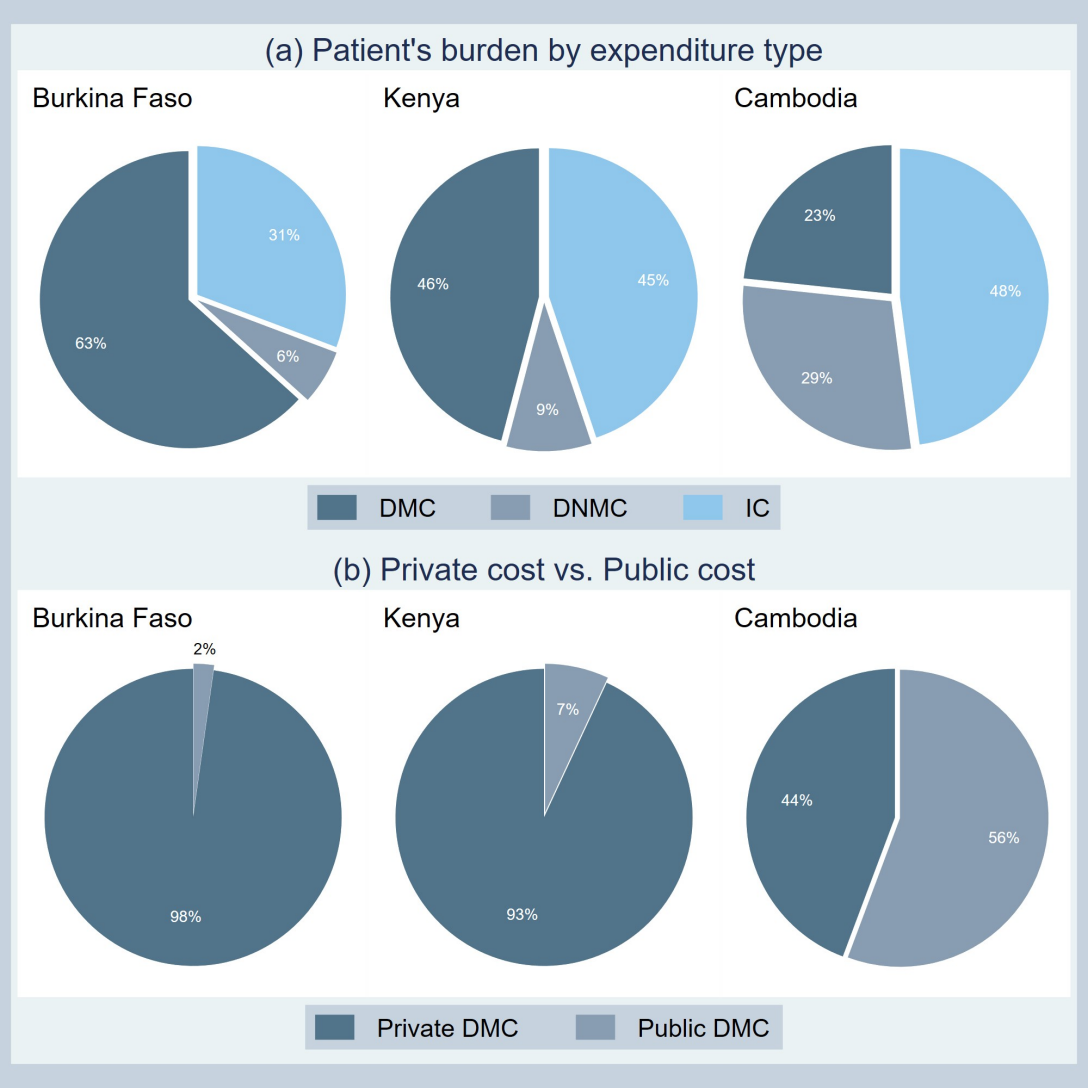
**The proportion of the economic burden for dengue fever by (a) expenditure type and (b) payer type.** The figure was standardized for direct comparisons among all six DVI countries. See Lee et al. for a standardized comparison with the first round countries [8].

Fig 2 demonstrates the proportions of the economic burden by cost component, as well as by expenditure payer. In Fig 2(A), while IC is the biggest burden for patients followed by DNMC and DMC in Cambodia, DMC accounts for the highest proportion of the patient’s private (out-of-pocket) burden in Burkina Faso and Kenya. Fig 2(B) compares the percentage contributions between patient’s private expenditure and public expenditure (i.e. insurance schemes, governmental subsidies, or other NGO aids, etc.). It is clear to see that compared to Cambodia, public contribution to the overall DMC is trivial in Burkina Faso and Kenya, meaning that the most of DMC burden has to be directly borne by patients. This finding is consistent with challenges on achieving Universal Health Coverage (UHC) in Africa [24,25].

The average economic burden of dengue fever is shown in Table 3. The total cost per dengue illness episode for inpatients converted by the official exchange rate is $26 and $134 in Burkina Faso and Cambodia, respectively. For outpatients, the average economic burden per dengue illness episode is estimated to be $13 in Burkina Faso and $23 in Kenya. After taking into account both private and public expenditure, the DMC component appears to be the biggest burden among the three major cost items in Burkina Faso and Kenya, whereas IC still remains the most significant contributor for the overall burden in Cambodia. The average cost per day ranges from $2 for outpatient in Burkina Faso to $15 for inpatient in Cambodia. The economic burden of dengue fever was also presented after adjusting the costs by the RCCs. While the total cost per dengue illness episode went up after the adjustment in Burkina Faso and Cambodia, this was the opposite in Kenya. The estimate in Cambodia shows the biggest change between the official exchange rate and the PPP conversion factor. It is worth noting that the WHO-CHOICE project shows cost per bed day, as well as cost per outpatient visit by hospital level [26]. While direct comparisons may not be appropriate due to different cost components, study designs, and target diseases, the total cost per day shown in the current study may be considered as a similar cost category.

**Table 3:** Average economic burden of dengue fever per episode (US$ and I$ in 2016)^a^.

**Official exchange rate**^**c**^	**Burkina Faso**						**Kenya**						**Cambodia**					
	**Inpatient (n = 141)**			**Outpatient (n = 273)**			**Inpatient (n = 0)**			**Outpatient (n = 149)**			**Inpatient (n = 254)**			**Outpatient (n = 0)**		
	**USD**	**BT CI**^**b**^ **(lower, upper)**		**USD**	**BT CI** **(lower, upper)**		**USD**	**BT CI** **(lower, upper)**		**USD**	**BT CI** **(lower, upper)**		**USD**	**BT CI** **(lower, upper)**		**USD**	**BT CI** **(lower, upper)**	
Direct Medical Cost (DMC)	$18	$17	$20	$7	$6	$8	-	-	-	$11	$9	$13	$42	$38	$46	-	-	-
Direct Non-Medical Cost (DNMC)	$1	$1	$2	$1	$1	$1	-	-	-	$2	$2	$3	$35	$30	$41	-	-	-
Indirect Cost (IC)	$6	$4	$9	$5	$4	$7	-	-	-	$10	$7	$14	$58	$46	$73	-	-	-
Total Cost	$26	$23	$29	$13	$11	$15	-	-	-	$23	$19	$28	$134	$119	$152	-	-	-
Total Cost per Day	$4	$4	$5	$2	$2	$3	-	-	-	$3	$2	$3	$15	$14	$17	-	-	-
Total Cost (RCC adjustment)	$27	$24	$30	$13	$11	$15	-	-	-	$23	$19	$28	$176	$161	$195	-	-	-
**PPP**^**d**^	**Burkina Faso**						**Kenya**						**Cambodia**					
	**Inpatient (n = 141)**			**Outpatient (n = 273)**			**Inpatient (n = 0)**			**Outpatient (n = 149)**			**Inpatient (n = 254)**			**Outpatient (n = 0)**		
	**I$**	**BT CI** **(lower, upper)**		**I$**	**BT CI** **(lower, upper)**		**I$**	**BT CI** **(lower, upper)**		**I$**	**BT CI** **(lower, upper)**		**I$**	**BT CI** **(lower, upper)**		**I$**	**BT CI** **(lower, upper)**	
Direct Medical Cost (DMC)	$51	$47	$55	$19	$17	$22	-	-	-	$24	$19	$29	$122	$111	$134	-	-	-
Direct Non-Medical Cost (DNMC)	$4	$3	$5	$3	$2	$3	-	-	-	$5	$4	$6	$103	$88	$120	-	-	-
Indirect Cost (IC)	$18	$12	$26	$15	$12	$19	-	-	-	$22	$15	$31	$171	$135	$216	-	-	-
Total Cost	$73	$64	$82	$37	$32	$43	-	-	-	$51	$41	$61	$396	$351	$449	-	-	-
Total Cost per Day	$12	$11	$13	$7	$6	$8	-	-	-	$6	$5	$7	$46	$41	$51	-	-	-
Total Cost (RCC adjustment)	$76	$68	$86	$37	$32	$43	-	-	-	$50	$40	$60	$520	$474	$575	-	-	-

^a^ See Lee et al. for a standardized comparison with the first round countries [8]

^b^ Bootstrapping with the percentile method

^c^ Official exchange rate per US$: 593 (Burkina Faso), 101.5 (Kenya), 4,058.7 (Cambodia)

^d^ PPP rate per international $: 209.6 (Burkina Faso), 46.7 (Kenya), 1,376,6 (Cambodia)

**Fig 3:**
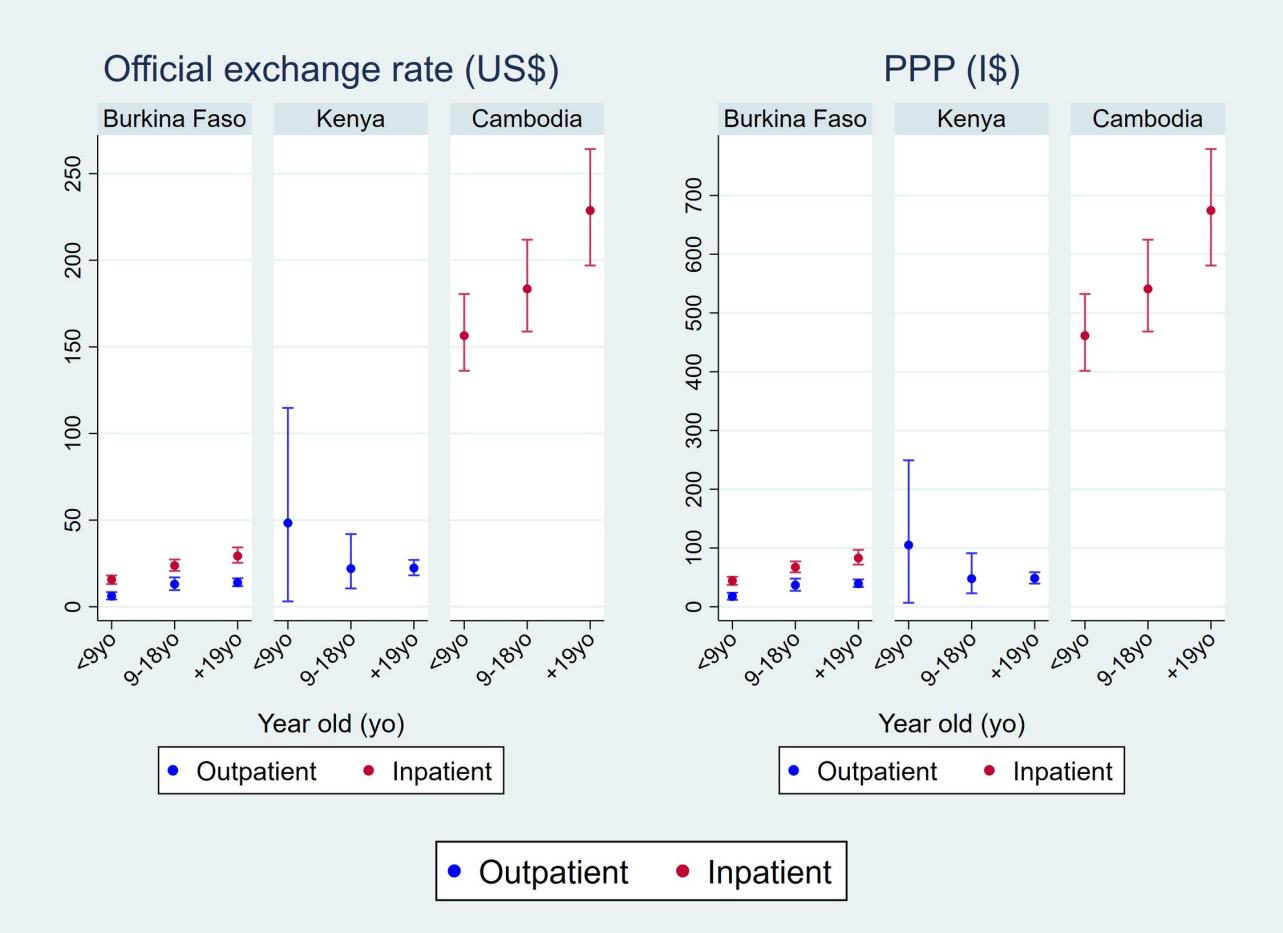
Economic burden by age group adjusted by the ratio of cost-to-charge (RCC).

Fig 3 demonstrates the average economic burden of dengue fever by age group. In Burkina Faso and Cambodia, the average cost increases from the younger age group to the older age group. On the other hand, the economic burden is higher for the youngest age group than for the other older age groups in Kenya. The high cost in the youngest group in Kenya was mainly derived from the additional private facility visit where patients paid much higher fees for medical services.

**Fig 4:**
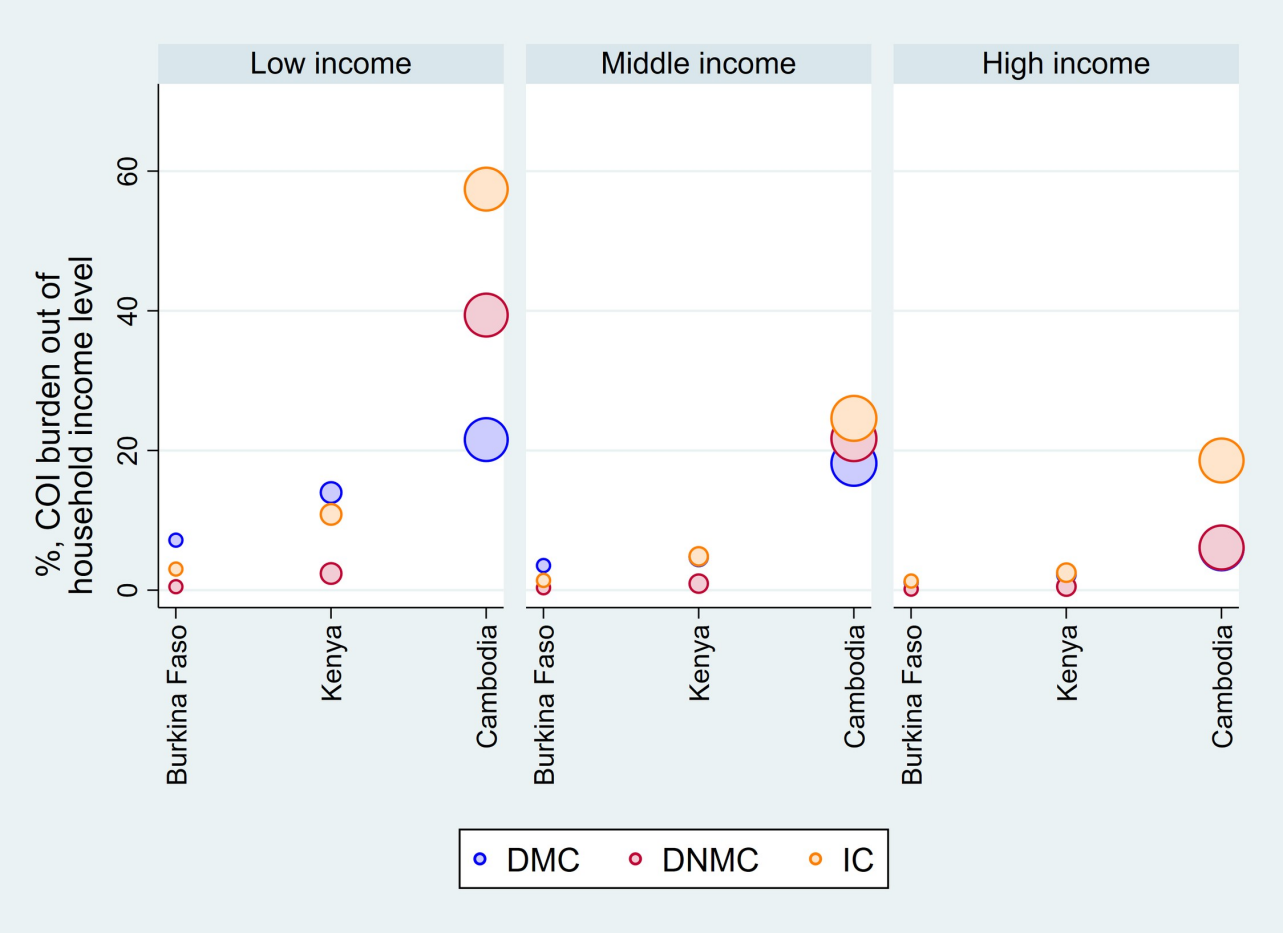
The proportion of the economic burden of dengue fever out of household income.

The patient’s private expenditure was estimated as a proportion of household’s monthly income and shown in Fig 4. For all three countries, the proportion of the private economic burden of dengue fever directly borne by patients was the highest in the low income group and decreased as moving towards the high income group. By country, the proportion of the private burden appeared to be relatively more significant in Cambodia compared to Burkina Faso and Kenya. In particular, the average direct expenditure due to dengue infection could be more than household’s monthly earning in the low income group in Cambodia.

## Discussion

The current study reports the most up-to-date estimates of the economic burden in Burkina Faso, Kenya, and Cambodia. In particular, having reviewed existing economic burden studies of dengue fever, our study is the first to understand the economic burden of dengue fever in Burkina Faso and Kenya based on primary data sources [17,27]. The study outcomes showed that the total economic burden of dengue fever is not trivial in all three countries. For inpatients, the average total cost per episode of dengue illness after the RCC adjustment was $26 in Burkina Faso and $134 in Cambodia. In the case of outpatients, the average cost per dengue episode was estimated to be $13 and $23 in Burkina Faso and Kenya, respectively.

Given that dengue has been prevalent for many years in Cambodia, several economic burden studies for dengue were previously done in this country. Four studies were identified at the time of this research [15,28–30]. Out of four, two studies estimated dengue cost-of-illness based on primary data sources [15,30]. Huy et al. reported $40 for inpatient which is lower than our estimate even after the inflation adjustment [30]. This is because Huy et al. only took into account private expenditure, whereas the current study included both private and public payments (i.e. health equity funds). On the other hand, Suaya at el. estimated the average cost of $115 per inpatient which is similar to the RCC-adjusted cost of the current study after the inflation adjustment [15]. Compared to the first round COI countries, the RCC adjusted total cost in Cambodia is similar to that in Thailand ($181) but lower than the costs in Vietnam ($213) and Colombia ($239). It is interesting to observe that the cost per inpatient converted using the PPP conversion factor in Cambodia is higher than those in Thailand and Colombia. Considering that purchasing power and parity is designed to equalize the purchasing power among different currencies, the economic burden of dengue in Cambodia is as significant as other dengue-endemic countries after taking into account differences in cost of living.

Overall, the total cost of illness for dengue fever was higher in Cambodia than in Burkina Faso and Kenya. In particular, the average cost per inpatient was much higher in Cambodia than in Burkina Faso although the average household income in Cambodia was lower than that in Burkina Faso. This was due to the following reasons: (1) the duration of illness was longer in Cambodia than in Burkina Faso, (2) while only 23% of the enrolled patients had sought medical care prior to coming to our study facilities in Burkina Faso, over 80% of the inpatients in Cambodia had done so increasing the overall spending, and (3) not many patients (approximately 30%) had caretakers during their illness in Burkina Faso, whereas all inpatients had caretakers in Cambodia, contributing to the significant increase in IC.

Nonetheless, the economic burden of dengue fever in the two African countries is not insignificant compared to the economic cost of malaria. Albeit by different methods, Beogo et al. reported $15.2 as the average cost of malaria in Burkina Faso [31]. Sicuri et al. estimated the economic costs of malaria in children in selected sub-Saharan countries and reported $11.2 for uncomplicated malaria and $51.9 for hospitalized malaria episodes in Kenya [32].

Some areas of uncertainty deserve attention. Despite the efforts to obtain financial reports from all four study facilities in Cambodia, the study team was not able to collect the financial report from one hospital out of four health facilities due to logistical issues. Thus, the RCC from the other health facility at the same level was applied assuming that the financial structure at the same level would not be substantially different. Nonetheless, additional information was obtained by implementing the medical service utilization form in Cambodia, and the bias was minimized. Similar to the first round COI study, the current COI study sites were limited to the areas where epidemiologic surveillance studies were carried out, thus caution must be exercised when interpreting the estimates beyond the study communities. In Burkina Faso, there was a dengue outbreak during the study period. This may have influenced healthcare practice in the health facilities, as well as health seeking behavior, particularly for children. However, the estimates in Burkina Faso may also be meaningful to understand the economic burden of dengue fever during an epidemic period. In Kenya, the study team tried to cover as many units within CPGH as possible but was unable to include inpatients due to logistical issues. Capital assets were not included in Cambodia and not depreciated in Burkina Faso and Kenya due to the lack of available information, thus the societal costs might be conservative in Cambodia and overestimated in the other two countries.

The standardized COI study was implemented in Burkina Faso, Kenya, and Cambodia. The selected study outcomes were presented in a similar way to the first-round COI study in order to facilitate comparisons across all six sites. In particular, the study findings clearly showed that the economic burden of dengue fever is significant not only in Cambodia but also in the two African countries. Given that the burden of dengue fever is relatively unknown in Africa, and that an increasing number of non-malaria fever patients have been reported [33,34], future research is urgently needed to have a better understanding of dengue disease burden in this region. For example, during the site selection period prior to implementing the current economic burden study in Kenya, health clinicians had repeatedly reported an increasing number of non-malaria fever patients during the mosquito season and were keen to understand the potential causes of the fever cases.

The first live attenuated, tetravalent dengue vaccine called Dengvaxia (CYD-TDV) became available in 2016. In addition, there are several second-generation vaccine candidates in the pipeline. Considering the broader availability of dengue vaccines in the future, it is critical to understand the societal benefits of vaccination and to develop sustainable financing plans taking into account competing health problems in the three countries. Along with more detailed epidemiological data (i.e. incidence rates) and evidence on the long-term behavior of a vaccine, the economic burden outcomes presented in the current study can be used to estimate more accurate vaccination benefits when conducting cost-effectiveness analyses of dengue vaccine interventions in the three countries in the future.

## Supporting information

S1 Text (DOCX)

S1 Table Additional descriptive statistics by age group. (DOCX)

S1 Fig Health facility visits before and after study enrollment. (DOCX)
