# Supplementary material for: A multi-country study of the economic burden of dengue fever based on patient-specific field surveys in Burkina Faso, Kenya, and Cambodia
Source: PLoS Negl Trop Dis. 2019 Feb 28;13(2):e0007164. doi: 10.1371/journal.pntd.0007164 (PMC6394908; doi:10.1371/journal.pntd.0007164)
Supplement: S1 Text — (DOCX) [file pntd.0007164.s001.docx]

#### 66BAppendix E-1. Cost-of-illness survey survey for Day 0 at the hospital

**Date of Interview: ___________ (dd/mm/yr) Interviewer code: _________________**

**Survey ID: __ __ __ __ __ __ __ __**

**Area/neighborhood _____________ Inpatient Outpatient**

###### Dengue Cost-of-Illness Questionnaire: Day 0

(Enumerator: Use this instrument to interview an adult (household head, parent, or caretaker) that is familiar with the patient and his or her treatment over the last few days. IF the patient is at least 18 years old, attempt to interview the patient, unless the patient requires a caretaker, e.g. elderly or has mental illness.)

Now I am going to ask you about how (your / your dependent’s) recent illness affected him/her and other people around him/her. As you already know, the health care providers at the study facility discovered that the patient was sick with dengue fever. First, I will ask about costs that your family incurred prior to visiting the treatment facility.

***Please think about what happened when you (or the patient) were (was) sick with dengue. If you (or the patient) were (was) sick with another disease before dengue, don’t tell me about that.***

1. How many days were you (or the patient) was sick with dengue before going to our health facility where he/she was diagnosed?

_____ Days

_____ (98) Other *(please specify)*

_____ (99) Don’t know

I am going to ask you about how much money was spent because of the illness. If you or the patient has medical insurance, please do not include his/her insurance payments in your responses. Also, do not report payments made by insurance company or program. I want to know how much you or your household had to spend over and above insurance payments.

1. Do you have insurance? If so, what type?

_____ (1) National hospital insurance fund

_____ (2) Other private insurance

_____ (3) Insurance provided by the employer other than option (1)

_____ (4) Free of charge (fee waived)

_____ (5) None

_____ (8) Other (please specify)

_____ (9) Don’t know

1. Could you please tell me your relationship to the patient? (*Enumerator: if are interviewing patient directly, check patient and skip this question*)

_____ (1) Patient (self)

_____ (2) Mother

_____ (3) Father

_____ (4) Brother

_____ (5) Sister

_____ (6) Grandfather

_____ (7) Grandmother

_____ (8) Uncle

_____ (9) Aunt

_____ (10) Cousin

_____ (98) Other *(please specify)*

**DIRECT COSTS - before coming to this hospital (today)**

First, I would like to know how this dengue episode affected you (or the patient) and your household.

1. Before coming to our health facility where he/she was diagnosed, did he/she receive treatment for this illness somewhere else?

_____ (1) Yes

_____ (2) No (End of the interview)

_____ (9) Don’t know (End of the interview)

**VISITS TO OTHER HEALTH CARE PROVIDERS**

For the patient who has received treatment for this illness from other health care facilities, please provide following details.

|  | Private  (a) | Public  (b) | Community health worker  (c) | Pharmacy / shop  (d) | Traditional  Healer  (e) | Others/ Self  (f) |
| --- | --- | --- | --- | --- | --- | --- |
| 1. No. of visits |  |  |  |  |  |  |
| 1. Total fee paid to the facility (for all visits) |  |  |  |  |  |  |
| 1. Medication |  |  |  |  |  |  |
| 1. Who paid for treatment (see list below) |  |  |  |  |  |  |
| 1. Transportation cost (patient + companions, round trip, all visits) |  |  |  |  |  |  |
| 1. Companion lodging |  |  |  |  |  |  |
| 1. Companion meals |  |  |  |  |  |  |
| 1. Special foods or drinks |  |  |  |  |  |  |
| 1. Extra payment to expedite treatment |  |  |  |  |  |  |
| 1. Other financial Costs   (specify which item) |  |  |  |  |  |  |

| Who paid for this visit? (TOTAL COST)  (1) Paid completely by insurance  (2) Paid completely out of pocket by patient or patient’s family  (3) Paid partly by insurance and partly out of pocket by patient or patient’s family  (4) Free of charge (fee waived)  (5) Other *(please specify)* __________________  (6) Don’t know |
| --- |
|  |

1. Was the patient hospitalized at any facility prior to visiting the study facility?

1. _____ yes

2. _____ no

9. _____ Don’t know

**CONCLUSION**

That completes our interview. Thank you very much for taking the time to answer my questions.

#### 67BAppendix E-2. Cost-of-illness survey household questionnaire (Day 10-14)

**Date of Interview: ___________ (dd/mm/yr) Interviewer code: _________________**

**Survey ID: __ __ __ __ __ __ __ __ Area/neighborhood _____________ The patient received all the treatment on the 1st day or the 2nd day during day 0** (tick one)

**1st day 2nd day**

**Has the person completed his/her log book (diary card)? Yes No**

**Dengue Cost-of-Illness Questionnaire: 10-14-day Follow-up**

(Enumerator: Use this instrument to interview an adult (patient, household head, parent, or caretaker) that is familiar with the patient and his or her treatment over the last few weeks. IF the patient is at least 18 years old, attempt to interview the patient, unless the patient requires a caretaker, e.g. elderly or has mental illness.)

Now I am going to ask you about how (your / your dependent’s) recent illness affected him/her and other people around him/her. As you already know, when the patient came to our health facility about 14 days ago, the clinic’s health care providers discovered that he/she was sick with dengue. I want to know about costs that have been incurred since the patient visited the hospital 10-14 days ago.

***Please refer to your log book (diary card) recorded when the patient was sick with dengue. If the patient was sick with another disease before dengue, don’t tell me about that. (Enumerator: If the respondent has their logbook before them, say, “Please check your logbook if it will help you remember.”)***

My records show that the patient went or was taken to our health facility about 10-14 days ago.

1. Is that correct?

_____ (1) Yes

_____ (2) No

_____ (9) Don’t know

Enumerator: The respondent should have responded “yes” to the last question. If they did not, ask if the patient is another household member or if there has been a mistake. If the problem cannot be resolved and the individual who was treated at the Treatment center cannot be identified, STOP the interview.

**DIRECT COSTS AT OUR HEALTH FACILITY**

I want to talk to you about the patient’s experience at our health facility where he/she was diagnosed and the costs related to that visit. Please do not consider the visits that occurred before or after that which you received the diagnosis.

1. What was the total amount your household had to spend to receive the treatment at our health facility where the patient was diagnosed, not including the cost of transportation, food, and lodging?

(If you or the patient has medical insurance, please do not include his/her insurance payments in your response. Also, do not report payments made by insurance companies or programs. This question asks only for direct medical costs: fees to a facility, medication. Note that if the patient has visited hospital for two days for the diagnosis of the 1st visit, include costs for the 2 days)

_____ KES

_____ (99999999) Don’t know

1. How long did it take to travel round trip and receive treatment?

(*Enumerator: Record respondents’ answer in either minutes OR hours, not both. In other words, if the one-way travel time was one and a half hours, record either 90 minutes or 1.5 hours*)

a. _____ Number of hours

b. _____ Number of minutes

_____ (99) Don’t know

1. What form of transportation was used?

_____ (1) Walk

_____ (2) Motorcycle

_____ (3) Car

_____ (4) Truck/bus

_____ (5) Bicycle

_____ (6) Tuktuk

_____ (7) Ambulance

_____ (8) Other *(please specify) ___________________*

1. Did any friends or family members accompany the patient when he/she sought care?

_____ (1) Yes

_____ (2) No (skip to 7)

_____ (9) Don’t know (skip to 7)

1. How many people accompanied the patient?

_____ (Number of people)

_____ (99) Don’t know

1. How much did transportation to our health facility cost, in money for the patient and all travel companions?

_____ KES

(99999999) Don’t know

1. How much did the patient and the people who accompanied the patient spend on food and/or lodging?

_____ KES

_____ (99999999) Don’t know

1. How many days has the patient felt sick or bad due to the symptoms of dengue fever since the first visit to our health facility?

_____ Number of days patient felt sick since last visit.

_____ (99) Don’t know

I am going to ask you about how much money was spent because of the patient’s illness. If the patient has medical insurance, please do not include his/her insurance payments in your responses. Also, do not report payments made by the patient insurance company or program. I want to know how much you or your household had to spend over and above the patient’s insurance payments.

**DIRECT COSTS**

First, I would like to know how this dengue episode affected the patient and your household.

Please, cross check with the patient’s diary card to see if answers are comparable. If not, verify and correct.

1. Besides when the patient went or was taken to the our health facility where he/she was diagnosed, did he/she receive treatment for this illness in the previous 10-14 days?

_____ (1) Yes

_____ (2) No *(skip to Q.22)*

_____ (9) Don’t know *(skip to Q.22)*

**VISITS TO OTHER HEALTH CARE PROVIDERS**

Where did the patient receive treatment after he/she went or was taken to our health facility where he/she was diagnosed? For each facility, we will ask you how many visits were made, the total cost, how treatment was paid for, and how much was paid for transportation and other expenses.

*Enumerator: ask the questions about each type of facility.*

|  | Private  (a) | Public  (b) | Community health worker  (c) | Pharmacy / shop  (d) | Traditional  Healer  (e) | Others/ Self  (f) | Our health facility (return visit)  (g) |
| --- | --- | --- | --- | --- | --- | --- | --- |
| 1. No. of visits |  |  |  |  |  |  |  |
| 1. Total fee paid to the facility (for all visits) |  |  |  |  |  |  |  |
| 1. Medication |  |  |  |  |  |  |  |
| 1. Who paid for treatment (see list below) |  |  |  |  |  |  |  |
| 1. Transportation cost (patient + companions, round trip, all visits) |  |  |  |  |  |  |  |
| 1. Companion lodging |  |  |  |  |  |  |  |
| 1. Companion meals |  |  |  |  |  |  |  |
| 1. Special foods or drinks |  |  |  |  |  |  |  |
| 1. Extra payment to expedite treatment |  |  |  |  |  |  |  |
| 1. Other financial Costs   (specify which item) |  |  |  |  |  |  |  |

| Who paid for this visit? (TOTAL COST)  (1) Paid completely by insurance  (2) Paid completely out of pocket by patient or patient’s family  (3) Paid partly by insurance and partly out of pocket by patient or patient’s family  (4) Free of charge (fee waived)  (5) Other *(please specify)* __________________  (6) Don’t know |
| --- |

1. Was the patient hospitalized at any facility since visiting our health facility?

_____ (1) Yes

_____ (2) No

_____ (9) Don’t know

**INDIRECT COSTS**

Now I want to ask you about the ways that the patient’s dengue illness affected him/her and others around him/her. When you answer these questions, please think about the entire time period during which the patient had dengue, starting with before the patient went or was taken to the clinic where he/she was diagnosed and until he/she completely recovered.

When someone is sick, this may affect the lives of other people in at least two important ways. First, sometimes when someone is sick and cannot do their usual activities, someone else has to do the patient's work/activities for them. Second, the sick person may need someone (perhaps a different person) to take care of them while they are sick. Thus one person may be doing the sick person's work and another person may be taking care of the sick person. I'm going to ask you about both of these kinds of helpers.

Sometimes people with dengue are so sick that they cannot perform any of their usual activities and they may have to stay in bed. They may even be unconscious.

Other people are not quite as sick. They can still perform *some* of their usual activities, but they cannot perform all of them, or they cannot perform their activities as well as normal. Now I want to ask you about the patient’s own experiences with dengue.

Please, cross check with the patient’s diary card to see if answers are comparable. If not, verify and correct.

1. In total, how many days has the patient been sick with dengue?

_____ Number of days

_____ (99) Don’t know

(*Enumerator; Check patient’s age. If patient is 5 years or less, then skip to Q. 44*

1. How many days was the patient completely unable to perform any of his/her usual activities (for the majority of one’s time)?

_____ Number of days

_____ (99) Don’t know

1. What would the patient have been doing if he/she had not been sick?

_____ (1) Going to school

_____ (2) Working on your own farm or self-employed

_____ (3) Housework (e.g., cooking, cleaning, taking care of children)

_____ (4) Working for a wage

_____ (5) Leisure time

_____ (8) Other *(please specify) ___________________*

1. How much does the patient normally earn for one day’s work? (if the patient does not earn any money, enter 0)

_____ KES

_____ (99999999) Don’t know

(*Enumerator: if the patient is paid monthly, please divide monthly salary by 24 and record amount above. If the patient is paid weekly, divide by 6 and report amount above*)

1. Did the patient lose any wages because of this illness?

_____ (1) Yes

_____ (2) No *(skip to Q 28)*

_____ (9) Don’t know *(skip to Q 28)*

1. In total, how many days’ wages did the patient lose because of this illness?

_____ Number of days

1. How many days was the patient able to perform *some* but not *all* of his/her usual activities?

_____ Number of days

_____ (99) Don’t know

1. How many days was the patient able to perform all his/her activities while sick?

_____ Number of days

_____ (99) Don’t know

(*Enumerator: Please check that the days of complete incapacitation, days of debilitation, and normal activity days add up to the total number of days ill.*

| Days of incapacitation (no activity while ill) (Q23) | + | Days of debilitation (some activity while ill) (Q28) | + | Days of normal activity while ill (Q29) | = | Total number of days ill *(Check that this is equal to number of sick days reported by respondent) (Q22)* |
| --- | --- | --- | --- | --- | --- | --- |
|  | + |  | + |  | = |  |

(*Enumerator: Please skip to question 44 if patient less than 13 years old.)*

**INDIRECT COSTS: SUBSTITUTE LABOR**

1. Did anyone perform the patient’s usual activities for him/her while he/she was sick with dengue fever?

_____ (1) Yes

_____ (2) No *(skip to Q. 44)*

_____ (9) Don’t know *(skip to Q. 44)*

1. How many people performed the patient’s usual activities while he/she was sick with dengue fever?

_____ Number of people

_____ (99) Don’t know

| Substitute Number | The patient’s relationship with the person who performed the patient’s usual activities while he/she was sick. | Is this person a member of your household?  (1) Yes  (2) No  (9) DK | Is this person an adult, teenager, or a child?  1=Adult (17+)  2=Teenager (12-16)  3=Child (Less than 12)  9=DK | How many hours/ days did he/she perform the patient’s usual activities?  *(Record No. of days and or number of hours as days/hours)*  (99) DK | Did this person perform:    1=All of patient’s activities  2=Some of patient’s activities  9=DK | Was this person paid to perform (patient name’s) activities?  1= *Yes*  2= No *(mark 0 in Q 38)*  9 =DK *(mark 99999999 in Q38)* | How much was this person paid per day?  DK 99999999 |
| --- | --- | --- | --- | --- | --- | --- | --- |
|  |  |  |  |  |  |  | KES |
| A |  |  |  |  |  |  |  |
| B |  |  |  |  |  |  |  |
| C |  |  |  |  |  |  |  |
| D |  |  |  |  |  |  |  |
| E |  |  |  |  |  |  |  |
| F |  |  |  |  |  |  |  |
| Substitute Number | Did this person who performed the patient’s activities cut back on his or her own usual activities?  1= *Yes*  2 = No *(skip to Q. 44)*  9 =DK *(skip to Q. 44)* | | How many hours/ days did this person who performed the patient’s activities cut back on his or her own duties?  *(Record No. of days and or number of hours as days/hours)*  99=DK | Was this person who performed the patient’s activities able to do:  1=Some of his/her own activities  2=None of his/her own activities  9 =DK | What would this person who performed the patient’s activities have been doing if he/she had not been performing the patient’s activities while he/she was sick?    1=Going to school  2 = Working on a farm  3 =Working at home  4 =Working for a wage  5 =Leisure time  8=Other *(specify)* | | 1. *(If he/she would have worked for a wage)* How much is this person who performed the patient’s activities normally paid for one day’s work? (for DK 99999999) |
|  |  |  |  |  |  |  | KES |
| A |  | |  |  |  | |  |
| B |  | |  |  |  | |  |
| C |  | |  |  |  | |  |
| D |  | |  |  |  | |  |
| E |  | |  |  |  | |  |
| F |  | |  |  |  | |  |

(*Enumerator: The following section should be asked about all patients, including those 5 or under*)

**INDIRECT COSTS: CARETAKING**

1. Was the patient so sick that someone had to cut back on his or her own usual activities for one or more days just to care for him/her? Care taking activities can include attending to the patient at home, accompanying the patient during trips to the hospital or other care providers or running errands for the patient such as picking up medication.

_____ (1) *Yes*

_____ (2) No *(skip to Q. 57)*

_____ (9) Don’t know *(skip to Q. 57)*

1. How many people cared for the patient while he/she was sick?

_____ Number of people

_____ (99) Don’t know

| Caretaker Number | The patient’s relationship with the person who cared for the patient while he/she was sick. | Is this person a member of your household?  (1) Yes  (2) No  (9) DK | Is this person an adult, teenager, or a child?  1=Adult(17+ years)  2=Teenager (12-16 years)  3=Child (<12 years)  9=DK | How many hours / days did he/she care for the patient?  *(Record No. of days and or number of hours as days/hours)*  (99) DK | Was this person paid to care for the patient?  1= *Yes*  2= No *(mark 0 in Q 51)*  9= DK *(mark 99999999 in Q51)* | How much was this person paid per day to care for the patient? (DK= 99999999) |
| --- | --- | --- | --- | --- | --- | --- |
|  |  |  |  |  |  | KES |
| A |  |  |  |  |  |  |
| B |  |  |  |  |  |  |
| C |  |  |  |  |  |  |
| D |  |  |  |  |  |  |
| E |  |  |  |  |  |  |
| F |  |  |  |  |  |  |

| Caretaker Number | Did this person who cared for the patient cut back on his or her own usual activities?  1= *Yes*  2 = No *(skip to Q. 57)*  9 =DK *(skip to Q. 57)* | How many hours / days did this person who cared for the patient cut back on his or her own duties?  *(Record No. of days and or number of hours as days/hours)*  99 =DK | Was this person who cared for the patient able to do:  1=Some of his/her own activities  2=None of his/her own activities  9=DK | What would this person who cared for the patient have been doing if he/she had not been caring for the patient while he/she was sick?  1=Going to school  2 = Working on a farm  3 =Working at home  4 =Working for a wage  5 =Leisure time  8=Other *(specify)* | 1. *(If he/she would have worked for a wage)* How much is this person who cared for the patient normally paid for one day’s work?   (DK=99999999) |
| --- | --- | --- | --- | --- | --- |
|  |  |  |  |  | KES |
| A |  |  |  |  |  |
| B |  |  |  |  |  |
| C |  |  |  |  |  |
| D |  |  |  |  |  |
| E |  |  |  |  |  |
| F |  |  |  |  |  |

1. Did your household have to borrow money from anyone in order to pay for the patient treatment of this illness?

_____ (1) Yes

_____ (2) No *(skip to Q 59)*

_____ (9) Don’t know *(skip to Q 59)*

1. Who did your household borrow money from?

_____ (1) Family member

_____ (2) Friend

_____ (3) Informal money lender

_____ (4) Bank/Credit Union

_____ (5) Other

1. Did your household have to sell any items in order to raise money to pay for the patient treatment of this illness?

_____ (1) Yes

_____ (2) No

_____ (9) Don’t know

**KNOWLEDGE AND PREVIOUS EXPERIENCE WITH DENGUE**

1. Please tell me all the ways that you believe dengue is transmitted. (*Enumerator: Do not read list. Check all that apply.).*

|  | a. | Mosquitoes |
| --- | --- | --- |
|  | b. | Bad weather |
|  | c. | Contact with infected family members |
|  | d. | Drinking poor quality wáter |
|  | e. | Standing water/open water storage containers |
|  | f. | Other *(please specify) ____________________* |
|  | g. | Don’t know |

1. If someone is sick with dengue, what do you believe are the best ways to cure this person? (*Enumerator: Do not read list. Check all that apply.).*

|  | a. | No treatment is necessary |
| --- | --- | --- |
|  | b. | Antibiotics *(please specify) __________________* |
|  | c. | Taking other medicine *(please specify) ___________________* |
|  | d. | Receiving treatment at the hospital |
|  | e. | Drinking a lot of clean wáter |
|  | f. | Injections |
|  | g. | Changing one’s diet |
|  | h. | Cleaning house, environment or body |
|  | i. | Religious healing |
|  | j. | Herbal medicines |
|  | k. | Other *(please specify) _________________* |
|  | l. | Don’t know |

1. What are the best ways to avoid getting dengue? (*Enumerator: Do not read list. Check all that apply.).*

|  | a. | There is no way to prevent dengue |
| --- | --- | --- |
|  | b. | Make sure all water storage containers have lids |
|  | c. | Control in house water quality |
|  | d. | Work with community to reduce/cover open water containers sewers |
|  | e. | Maintain clean and proper latrines |
|  | f. | Maintain adequate waste disposal |
|  | g. | Avoid overwatering plants or garden |
|  | h. | Clean the house or environment |
|  | i. | Avoid contact with patients or carriers |
|  | j. | Get vaccinated |
|  | k. | Take preventative medicine *(please specify) __________________* |
|  | l. | Other *(please specify) ___________________* |
|  | m. | Don’t know |

1. Who does dengue affect more seriously? (*Enumerator: check only one.*)

_____ (1) Adults (18+ years)

_____ (2) School-age children (6-17 years)

_____ (3) Preschool children (1-5 years)

_____ (4) Infants (<1 year)

_____ (5) Elderly (55+ years)

_____ (6) Equally serious to all age groups

_____ (7) Not serious in any age group

_____ (9) Don’t know

**(CONSIDER ONLY LAST 3-YEAR DATA)**

1. Has the patient had dengue before?

_____ (1) *Yes*

_____ (2) No *(skip to Q 66)*

_____ (9) Don’t know *(skip to Q 66)*

1. How many times has the patient had dengue before?

_____ Number of times

_____ (99) Don’t know

1. Has anyone in your household ever died of dengue?

_____(1) Yes

_____(2) No *(skip to Q 69)*

_____(9) Don’t know *(skip to Q 69*)

| 1. How old were they when they died? | 1. What year did they die? |
| --- | --- |
| A. | A. |
| B. | B. |
| C. | C. |

1. Has any adult now living in your household had dengue before and recovered?

_____ (1) Yes

_____ (2) No *(skip to Q 72)*

_____ (9) Don’t know *(skip to Q 72)*

1. How many adults now living in this household have had dengue before and recovered?

_____ Number of adults who have had dengue

_____ (99) Don’t know *(skip to Q 72)*

(*Enumerator: Complete the table by asking the respondent the questions in the top row.)*

| 1. How many times has this adult had dengue and recovered? |
| --- |
| a. |
| b. |
| c. |

1. Have any other children now living in your household had dengue before and recovered?

_____ (1) *Yes*

_____ (2) No *(skip to q 75)*

_____ (9) Don’t know *(skip to Q 75)*

1. How many other children now living in this household have had dengue before and recovered?

_____ Number of children who have had dengue

_____ (99) Don’t know *(skip to Q. 75)*

(*Enumerator: Complete the table by asking the respondent the questions in the top row).*

| 1. How many times has this child had dengue and recovered? |
| --- |
| a. |
| b. |
| c. |

1. Do you know anyone outside the family that has died of dengue?

_____ (1) *Yes*

_____ (2) No *(skip to Q.77)*

_____ (9) Don’t know *(skip to Q.77)*

1. If yes, how many people outside your family do you know who have died of dengue?

No. of non-family members respondent knows who have died of dengue ___________

**AVERTIVE BEHAVIOR**

1. Do member of this household have done anything special or spend any money on anything to specifically avoid getting infected with dengue, including use of bed nets, screen, mosquito repellent, use of treated sand, covering water storage containers or prevention of standing water near the home?

(1) _____ Yes

(2) _____ No *(skip to Q 85)*

(9) _____ Don’t know *(skip to Q 85)*

1. Does your family take any of the following precautions to avoid dengue fever? (*Enumerator: read responses, more than one response permitted)*

(a) _____ Cover water containers

(b) _____ Remove/drain items where water may collect (e.g., tires, buckets)

(c) _____ Electric racket

(d) _____ Use chemical sprays/repellent

(e) _____ Avoid areas with dengue

(f) _____ Use of mosquito/bed net

(g) _____ Use of window screen

(h)______ Change water in flower pots

(i) _____ Other (please specify) _____________________

(j) _____ Don’t know/not sure

1. How often do household members use chemical mosquito spray or treated sand?

(1) _____ All the time

(2). _____ During dengue season

(3) _____ During dengue outbreaks

(4) _____ Never

(8) _____ Other *(please specify) ____________________*

(9) _____ Don’t know

1. Dengue vector control activities may only be undertaken during the peak dengue season (peak season). How many months or weeks are dengue vector control activities undertaken (i.e. insecticide, mosquito repellent, special containers, mosquito net, etc.)? (please report weeks or months, not both)

a. _____ Weeks

b. _____ Months

_____ (99) Don’t know

1. For this household, how many hours per week are spent on dengue vector control activities during the dengue season?

_____ Hours

_____ (99) Don’t know

1. Do you cover your water storage containers?

(1) _____ Yes

(2) _____ No

(9) _____ Don't know

1. Do you try to prevent standing water near your household?

(1) _____ Yes

(2) _____ No

(9) _____ Don't know

1. How much money per month does the household spend on dengue vector control (e.g., insecticide, mosquito repellent, special containers) during peak dengue season(peak season )?

_____ KES (*skip to End of section)*

_____ (99999999) Don’t know (*skip to End of section)*

1. *If members of the household do NOT perform dengue vector control activities, ASK:* Why not?

(1) _____ Not effective

(2) _____ Too time consuming/inconvenient

(3) _____ Not applicable

(4) _____ Not aware of it

(5) _____ Worried about health concerns (spray or sand)

(6) _____ Dengue not a problem here

(8) _____ Other *(please specify) ____________________*

(9) _____ Don’t know

**Socioeconomic questions**

Enumerator: Please ask these questions in below table for the respondent on behalf of his/her household**.**

| List | What class of school did you complete or what degrees have you received? | Occupation | Earnings per month  (KES Per month)  DK- 99999999 |
| --- | --- | --- | --- |
| Patient |  |  |  |
| Respondent(ifdifferent from patient) |  |  |  |
| Head of household |  |  |  |

| 86.  Education level | 87.  Occupation List | |
| --- | --- | --- |
| 1= Have never attended school  2= 1 to 5 years of school  3= 6 to 9 years of school  4= 10 to 12 years of school  5= Vocational studies/college diploma  6= University/college  7= Post-graduate studies  8= Professional course  9= Religious education  10=Informal education  97=Others (Specify) ________  98=Refuse to answer  99=Don’t know | 1= Student  2= Retired  3= Housewife  4= Unemployed  5= Professional  6= Unskilled office worker  7= Owner of business  8= Unskilled manual worker  9= Skilled manual worker | 10= Street seller  11= Driver  12= Public servant  13= Small business  14= Fisherman  15= Service worker (egg. driver, servant, cook, hotel or restaurant worker)  97= Other (Specify)  98=Refuse to answer  99=Don’t know |

1. How much does your household earn per month?

___________ KES /month

(99999999)____ Don’t know/not sure

(98888888)____ No response

1. How much does your household spend during an average month?

___________ KES /month

(99999999)____ Don’t know/not sure

(98888888)____ No response

1. Can you read a newspaper if we give you a newspaper?

(1)______ Yes, Easily

(2)______ Yes, with difficulty

(3)______ No, cannot read a newspaper.

**Household Assets**

1. Could you tell me whether or not someone in your household owns a(n) (category name)?

|  | (yes) | (no) |  |
| --- | --- | --- | --- |
|  |  |  | a.Cot/bed |
|  |  |  | b.Cabinet (Cupboard) |
|  |  |  | c.Car |
|  |  |  | d.Pick-up truck |
|  |  |  | e. Washing machine |
|  |  |  | f.Radio |
|  |  |  | g.Color TV |
|  |  |  | h.Refrigerator |
|  |  |  | i. Mobile phone |
|  |  |  | j. Air Conditioner |
|  |  |  | k.Internet |
|  |  |  | l.Computer |

**Electricity**

1. How much was your household’s own electricity bill last month? Or, if you share the bill, how much was your share?

__________ Household’s own share of electricity bill (KES)

(66666666) _____ We pay no monthly charge for electricity / Electricity is included in the rent

(99999999) _____ Don’t know/not sure

(98888888) _____ No response

94. Could you please tell me your relationship to the patient?

1. Patient
2. Mother
3. Father
4. Brother
5. Sister
6. Grandfather
7. Grandmother
8. Uncle
9. Aunt
10. Cousin

(98) Other *(please specify) ___________________*

95. Is the patient still sick with dengue fever:

(1) _____ Yes (Remind patient of logbook instructions and schedule another interview at

the patient’s house for day 28)

(2) _____ No (End)

**CONCLUSION**

That completes our interview. Thank you very much for taking the time to answer my questions

#### 68BAppendix E-3. Cost-of-illness survey household questionnaire (Day 28)

**Date of Interview: ___________ (dd/mm/yr) Interviewer code: _________________**

**Survey ID: __ __ __ __ __ __ __ __ Area/neighborhood _____________**

**DENGUE Cost-of-Illness Questionnaire: 28-day Follow-up**

(Enumerator: Use this instrument to interview an adult (patient, household head, parent, or caretaker) that is familiar with the patient and his or her treatment over the last few weeks. IF the patient is at least 18 years old, attempt to interview the patient, unless the patient requires a caretaker, e.g. elderly or has mental illness.)

Now I am going to ask you about how (your / your dependent’s) recent illness affected him/her and other people around him/her. As you already know, when the patient came to our health facility about 28 days ago, the clinic’s health care providers discovered that he/she was sick with dengue. I want to know about costs that have been incurred since the last interview about 10-14 days ago.

***Please refer to your log book (diary card) recorded when the patient was sick with dengue. If the patient was sick with another disease before dengue, don’t tell me about that. (Enumerator: If the respondent has their logbook before them, say, “Please check your logbook if it will help you remember.”)***

My records show that the patient went or was taken to our health facility about 28 days ago.

1. Is that correct?

_____ (1) Yes

_____ (2) No

_____ (9) Don’t know

Enumerator: The respondent should have responded “yes” to the last question. If they did not, ask if the patient is another household member or if there has been a mistake. If the problem cannot be resolved and the individual who was treated at the Treatment center cannot be identified, STOP the interview.

1. How many days has the patient felt sick or bad due to the symptoms of dengue fever since then?

_____ Number of days patient felt sick since last visit.

_____ (99) Don’t know

I am going to ask you about how much money was spent because of the patient’s illness. If the patient has medical insurance, please do not include his/her insurance payments in your responses. Also, do not report payments made by the patient insurance company or program. I want to know how much you or your household had to spend over and above the patient’s insurance payments.

**DIRECT COSTS (Approximately Days 15-28)**

1. Since we talked the last time, has the patient gone or been taken anywhere for treatment of dengue fever?

____ (1) Yes

____ (2) No*(skip to q 15)*

____ (9) Don’t know *(skip to q 15)*

**VISITS TO OTHER HEALTH CARE PROVIDERS**

***Enumerator: ask the questions about each type of facility.***

|  | Private  (a) | Public  (b) | Community health worker  (c) | Pharmacy / shop  (d) | Traditional  Healer  (e) | Others/ Self  (f) | Our health facility (return visit)  (g) |
| --- | --- | --- | --- | --- | --- | --- | --- |
| 1. No. of visits |  |  |  |  |  |  |  |
| 1. Total fee paid to the facility (for all visits) |  |  |  |  |  |  |  |
| 1. Medication |  |  |  |  |  |  |  |
| 1. Who paid for treatment (see list below) |  |  |  |  |  |  |  |
| 1. Transportation cost (patient + companions, round trip, all visits) |  |  |  |  |  |  |  |
| 1. Companion lodging |  |  |  |  |  |  |  |
| 1. Companion meals |  |  |  |  |  |  |  |
| 1. Special foods or drinks |  |  |  |  |  |  |  |
| 1. Extra payment to expedite treatment |  |  |  |  |  |  |  |
| 1. Other financial Costs   (specify which item) |  |  |  |  |  |  |  |

| Who paid for this visit? (TOTAL COST)  (1) Paid completely by insurance  (2) Paid completely out of pocket by patient or patient’s family  (3) Paid partly by insurance and partly out of pocket by patient or patient’s family  (4) Free of charge (fee waived)  (5) Other *(please specify)* __________________  (6) Don’t know |
| --- |

1. Was the patient hospitalized at any facility since visiting our health facility?

_____ (1) Yes

_____ (2) No

_____ (9) Don’t know

**INDIRECT COSTS**

Now I want to ask you about the ways that the patient’s dengue illness affected him/her and others around him/her. When you answer these questions, please think about the entire time period during which the patient had dengue. When you answer these questions, please consider the time period starting with before the patient went or was taken to the clinic where he/she was diagnosed and ending today.

When someone is sick, this may affect the lives of other people in at least two important ways. First, sometimes when someone is sick and cannot do their usual activities, someone else has to do the patient's work/activities for them. Second, the sick person may need someone (perhaps a different person) to take care of them while they are sick. Thus one person may be doing the sick person's work and another person may be taking care of the sick person. I'm going to ask you about both of these kinds of helpers.

Please, cross check with the patient’s diary card to see if answers are comparable. If not, verify and correct.

1. In total, how many days has the patient been sick with dengue?

_____ Number of days

_____ (99) Don’t know

(*Enumerator; Check patient’s age. If patient is 5 years or less, then skip to Q. 37*

1. How many days was the patient completely unable to perform any of his/her usual activities?

_____ Number of days

_____ (99) Don’t know

1. What would the patient have been doing if he/she had not been sick?

_____ (1) Going to school

_____ (2) Working on a farm

_____ (3) Working at home

_____ (4) Working for a wage

_____ (5) Leisure time

_____ (8) Other *(please specify) ___________________*

1. *(If they would have worked for a wage)* How much does the patient normally earn for one day’s work?

_____ KES

_____ (99999999) Don’t know

(*Enumerator: if the patient is paid monthly, please divide monthly salary by 24 and record amount above. If the patient is paid weekly, divide by 6 and report amount above*)

1. Did the patient lose any wages because of this illness?

_____ (1) Yes

_____ (2) No *(skip to Q 21)*

_____ (9) Don’t know *(skip to Q 21)*

1. In total, how many days’ wages did the patient lose because of this illness?

_____ Number of days

1. How many days was the patient able to perform *some* but not *all* of his/her usual activities?

_____ Number of days

_____ (99) Don’t know

1. How many days was the patient able to perform all his/her activities while sick?

_____ Number of days

_____ (99) Don’t know

(*Enumerator: Please check that the days of complete incapacitation, days of debilitation, and normal activity days add up to the total number of days ill.*

| Days of incapacitation (no activity while ill) Q16 | + | Days of debilitation (some activity while ill) Q21 | + | Days of normal activity while ill Q22 | = | Total number of days ill *(Check that this is equal to number of sick days reported by respondent) Q15* |
| --- | --- | --- | --- | --- | --- | --- |
|  | + |  | + |  | = |  |

**INDIRECT COSTS: SUBSTITUTE LABOR**

1. Did anyone perform the patient usual activities for him/her while he/she was sick with dengue fever?

_____ (1) Yes

_____ (2) No *(skip to Q. 37)*

_____ (9) Don’t know *(skip to Q. 37)*

1. How many people performed the patient’s usual activities while he/she was sick with dengue fever?

_____ Number of people

_____ (99) Don’t know

| Substitute Number | The patient’s relationship with the people who performed the patient’s usual activities while he/she was sick. | Is this person a member of your household?  (1) Yes  (2) No  (9) DK | Is this person an adult, teenager, or a child?  1=Adult (17+)  2=Teenager (12-16)  3=Child (Less than 12)  9=DK | How many hours / days did he/she perform the patient’s usual activities?  *(Record No. of days and or number of hours as days/hours)*  (99) DK | Did this person perform:    1=All of patient’s activities  2=Some of patient’s activities  9=DK | Was this person paid to perform (patient name’s) activities?  1= Yes  2= No *(mark 0 in Q 31)*  9 =DK *(mark 99999999 in Q 31)* | How much was this person paid per day?  DK-99999999 |
| --- | --- | --- | --- | --- | --- | --- | --- |
|  |  |  |  |  |  |  | KES |
| A |  |  |  |  |  |  |  |
| B |  |  |  |  |  |  |  |
| C |  |  |  |  |  |  |  |
| D |  |  |  |  |  |  |  |
| E |  |  |  |  |  |  |  |
| Substitute Number | Did this person who performed the patient’s activities cut back on his or her own usual activities?  1= *Yes*  2 = No *(skip to Q. 37)*  9 =DK *(skip to Q. 37)* | | How many hours / days did this person who performed the patient’s activities cut back on his or her own duties?  *(Record No. of days and or number of hours as days/hours)*  99 =DK | Was this person who performed the patient’s activities able to do:  1=Some of his/her own activities  2=None of his/her own activities  9 =DK | What would this person who performed the patient’s activities have been doing if he/she had not been performing the patient’s activities while he/she was sick?    1=Going to school  2 = Working on a farm  3 =Working at home  4 =Working for a wage  5 =Leisure time  8=Other *(specify)* | | 1. *(If he/she would have worked for a wage)* How much is this person who performed the patient’s activities normally paid for one day’s work? |
|  |  |  |  |  |  |  | KES  DK-99999999 |
| A |  | |  |  |  | |  |
| B |  | |  |  |  | |  |
| C |  | |  |  |  | |  |
| D |  | |  |  |  | |  |
| E |  | |  |  |  | |  |
| F |  | |  |  |  | |  |

**INDIRECT COSTS: CARETAKING**

1. Was the patient so sick that someone had to cut back on his or her own usual activities for one or more days just to care for him/her?

_____ (1) Yes

_____ (2) No *(skip to Q. 50)*

_____ (9) Don’t know *(skip to q. 50)*

1. How many people cared for the patient while he/she was sick?

_____ Number of people

_____ (99) Don’t know

| Caretaker Number | The patient’s relationship with the people who cared for the patient while he/she was sick. | Is this person a member of your household?  (1) Yes  (2) No  (9) DK | Is this person an adult, teenager, or a child?  1=Adult  2=Teenager  3=Child  9=DK | How many hours / days did he/she care for the patient?  *(Record No. of days and or number of hours as days/hours)*  (99) DK | Was this person paid to care for the patient?  1= *Yes*  2= No *(mark 0 in Q 44)*  9 =DK *(mark 99999999 in Q44)* | How much was this person paid per day to care for the patient? |
| --- | --- | --- | --- | --- | --- | --- |
|  |  |  |  |  |  | KES  DK-99999999 |
| A |  |  |  |  |  |  |
| B |  |  |  |  |  |  |
| C |  |  |  |  |  |  |
| D |  |  |  |  |  |  |
| E |  |  |  |  |  |  |
| F |  |  |  |  |  |  |

| Caretaker Number | Did this person who cared for the patient cut back on his or her own usual activities?  1= Yes  2 = No *(skip to Q. 50)*  9 =DK *(skip to Q. 50)* | How many hours / days did this person who cared for the patient cut back on his or her own duties?  *(Record No. of days and or number of hours as days/hours)*  99) =DK | Was this person who cared for the patient able to do:  1=Some of his/her own activities  2=None of his/her own activities  9 =DK | What would this person who cared for the patient have been doing if he/she had not been caring for the patient while he/she was sick?  1=Going to school  2 = Working on a farm  3 =Working at home  4 =Working for a wage  5 =Leisure time  8=Other *(specify)* | 1. *(If he/she would have worked for a wage)* How much is this person who cared for the patient normally paid for one day’s work? |
| --- | --- | --- | --- | --- | --- |
|  |  |  |  |  | KES  DK-99999999 |
| A |  |  |  |  |  |
| B |  |  |  |  |  |
| C |  |  |  |  |  |
| D |  |  |  |  |  |
| E |  |  |  |  |  |
| F |  |  |  |  |  |

1. Did your household have to borrow money from anyone in order to pay for the patient treatment of this illness?

_____ (1) *Yes*

_____ (2) No *(skip to Q 52)*

_____ (9) Don’t know *(skip to Q 52)*

1. Who did your household borrow money from?

_____ (1) Family member

_____ (2) Friend

_____ (3) Informal money lender

_____ (4) Bank/Credit Union

_____ (8) Other

1. Did your household have to sell any items in order to raise money to pay for the patient treatment of this illness?

_____ (1) Yes

_____ (2) No

_____ (9) Don’t know

1. Could you please tell me your relationship to the patient?
2. Patient
3. Mother
4. Father
5. Brother
6. Sister
7. Grandfather
8. Grandmother
9. Uncle
10. Aunt
11. Cousin

_____ (98) Other *(please specify) ___________________*

**CONCLUSION**

That completes our interview. Thank you very much for taking the time to answer my question
