# Supplementary material for: A multi-country study of the economic burden of dengue fever based on patient-specific field surveys in Burkina Faso, Kenya, and Cambodia
Source: PLoS Negl Trop Dis. 2019 Feb 28;13(2):e0007164. doi: 10.1371/journal.pntd.0007164 (PMC6394908; doi:10.1371/journal.pntd.0007164)
Supplement: S1 Table — (DOCX) [file pntd.0007164.s002.docx]

**S1 Table. Additional descriptive statistics by age group**

| **Country** | **Item** | **<9yo** | **(SD)** | **9-18yo** | **(SD)** | **+19yo** | **(SD)** |
| --- | --- | --- | --- | --- | --- | --- | --- |
| Burkina Faso | Age distribution (%) | 7.5% | - | 16.7% | - | 75.8% | - |
|  | Number of sick days (outpatient) | 5.3 | (1.4) | 5.8 | (1.8) | 5.7 | (2.0) |
|  | Number of sick days (inpatient) | 6.6 | (1.8) | 5.8 | (1.7) | 6.2 | (2.4) |
|  | Number of facility visits (outpatient) | 1.2 | (0.4) | 1.2 | (0.5) | 1.2 | (0.5) |
|  | Number of facility visits (inpatient) | 1.0 | (0.0) | 1.4 | (0.6) | 1.3 | (0.5) |
| Kenya | Age distribution (%) | 2.0% | - | 12.8% | - | 85.2% | - |
|  | Number of sick days (outpatient) | 8.0 | (2.0) | 8.2 | (4.3) | 8.1 | (3.1) |
|  | Number of sick days (inpatient) | - | - | - | - | - | - |
|  | Number of facility visits (outpatient) | 2.0 | (1.0) | 1.9 | (1.2) | 1.8 | (0.9) |
|  | Number of facility visits (inpatient) | - | - | - | - | - | - |
| Cambodia | Age distribution (%) | 39.8% | - | 52.4% | - | 7.9% | - |
|  | Number of sick days (outpatient) | - | - | - | - | - | - |
|  | Number of sick days (inpatient) | 8.5 | (2.8) | 8.6 | (2.6) | 9.1 | (2.3) |
|  | Number of facility visits (outpatient) | - | - | - | - | - | - |
|  | Number of facility visits (inpatient) | 2.6 | (1.5) | 3.1 | (1.7) | 3.5 | (2.1) |
