# Supplementary material for: A multi-country study of the economic burden of dengue fever based on patient-specific field surveys in Burkina Faso, Kenya, and Cambodia
Source: PLoS Negl Trop Dis. 2019 Feb 28;13(2):e0007164. doi: 10.1371/journal.pntd.0007164 (PMC6394908; doi:10.1371/journal.pntd.0007164)
Supplement: S1 Fig — (DOCX) [file pntd.0007164.s003.docx]

**S1 Figure. Health facility visits before and after study enrollment ^a^**


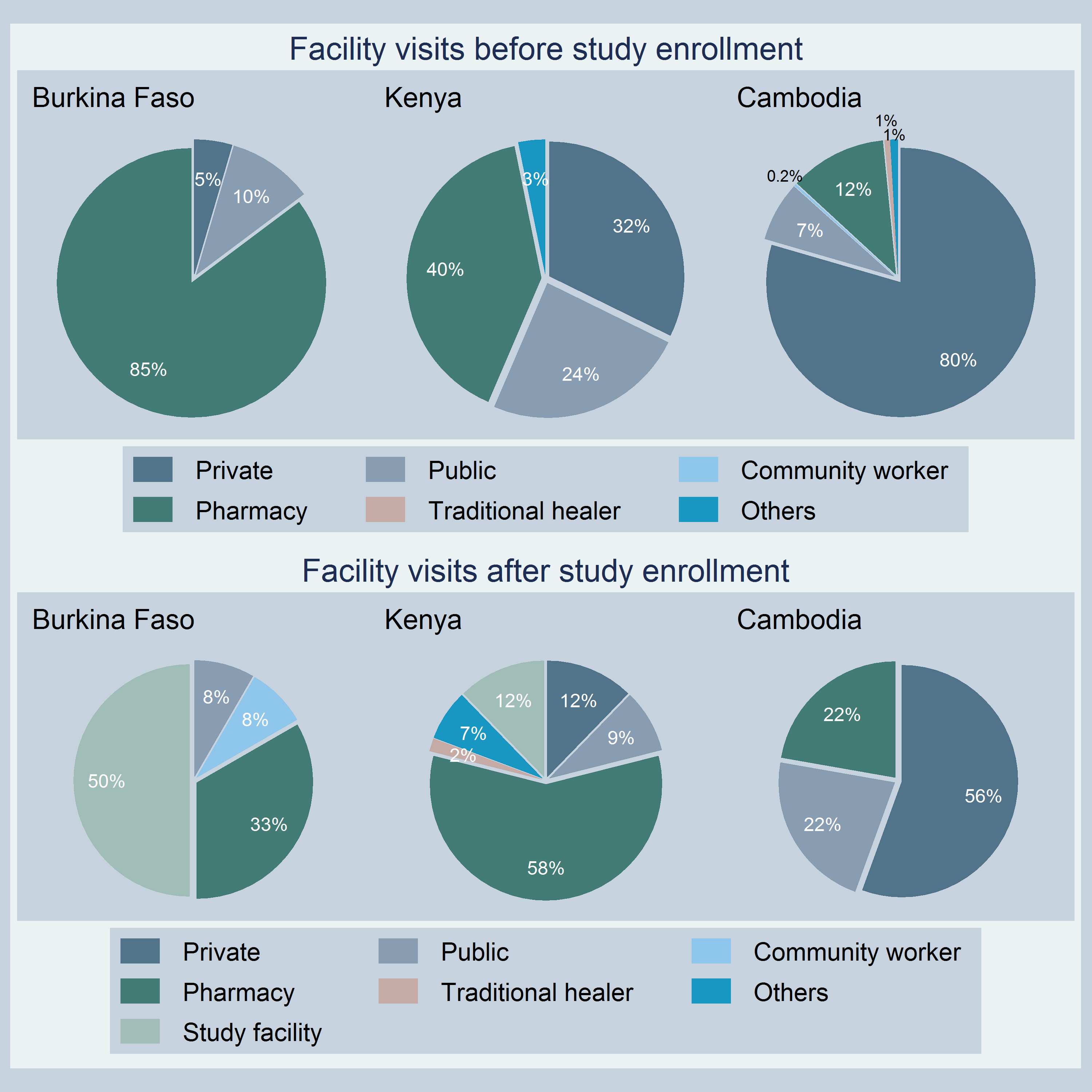


^a^ The percentages shown were for those who made at least 1 visit to one of the health facilities.
